# Comparative Application of Terminal Restriction Fragment Analysis Tools to Large-Scale Genomic Assays

**DOI:** 10.3390/ijms242417194

**Published:** 2023-12-06

**Authors:** Liliia R. Abdulkina, Inna A. Agabekian, Liia R. Valeeva, Olga S. Kozlova, Margarita R. Sharipova, Eugene V. Shakirov

**Affiliations:** 1 Institute of Fundamental Medicine and Biology, Kazan Federal University, Kazan 420008, Republic of Tatarstan, Russia; nigmatullinalili@mail.ru (L.R.A.); nuceq929@mail.ru (I.A.A.); valeeva@marshall.edu (L.R.V.); olga-sphinx@yandex.ru (O.S.K.); marsharipova@gmail.com (M.R.S.); 2 Department of Biological Sciences, College of Science, Marshall University, Huntington, WV 25701, USA; 3 Department of Biomedical Sciences, Joan C. Edwards School of Medicine, Marshall University, Huntington, WV 25755, USA

**Keywords:** telomerase, TeloTool, WALTER, telomere length, SNP, GWAS

## Abstract

The analysis of telomere length is an important component of many studies aiming to characterize the role of telomere maintenance mechanisms in cellular lifespan, disease, or in general chromosome protection and DNA replication pathways. Several powerful methods to accurately measure the telomere length from Southern blots have been developed, but their utility for large-scale genomic studies has not been previously evaluated. Here, we performed a comparative analysis of two recently developed programs, TeloTool and WALTER, for the extraction of mean telomere length values from Southern blots. Using both software packages, we measured the telomere length in two extensive experimental datasets for the model plant *Arabidopsis thaliana*, consisting of 537 natural accessions and 65 T-DNA (transfer DNA for insertion mutagenesis) mutant lines in the reference Columbia (Col-0) genotype background. We report that TeloTool substantially overestimates the telomere length in comparison to WALTER, especially for values over 4500 bp. Importantly, the TeloTool- and WALTER-calculated telomere length values correlate the most in the 2100–3500 bp range, suggesting that telomeres in this size interval can be estimated by both programs equally well. We further show that genome-wide association studies using datasets from both telomere length analysis tools can detect the most significant SNP candidates equally well. However, GWAS analysis with the WALTER dataset consistently detects fewer significant SNPs than analysis with the TeloTool dataset, regardless of the GWAS method used. These results imply that the telomere length data generated by WALTER may represent a more stringent approach to GWAS and SNP selection for the downstream molecular screening of candidate genes. Overall, our work reveals the unanticipated impact of the telomere length analysis method on the outcomes of large-scale genomic screens.

## 1. Introduction

The ends of linear eukaryotic chromosomes are protected by telomeres, evolutionarily conserved protein–DNA complexes that are involved in genome maintenance and the regulation of cellular lifespan. The critical functions of telomeres in chromosome protection were originally revealed by the classical studies of Barbara McClintock in maize [1,2]. The mechanism of chromosome end deprotection was later predicted by Alexey Olovnikov to involve the gradual attrition of telomere DNA over multiple cell divisions [3]. The approximate rate of telomere shortening in human somatic cells is 50–200 bp per cell division, mostly due to the intrinsic inability of conventional DNA polymerases to fully replicate linear chromosome ends [4]. When telomeres reach a critical length, they fail to bind enough telomere-associated proteins, ultimately contributing to the so-called Hayflick limit of cell proliferation [5,6]. The discovery of the repeated nature of eukaryotic telomeres [7] opened the door for the analysis of the telomere length, which, in various organisms, was found to be incredibly varied, ranging from as little as 300 base pairs in yeast [8] to as much as 160 kb in tobacco [9], while also varying up to 25-fold between different genotypes of the same species [10].

In recent years, many highly sensitive telomere length measurement assays have been developed, including Q-PCR, Q-FISH, STELA, and TeSLA, among others [11]. While most of these assays are very precise, many also have important drawbacks, including being very relative or expensive or requiring sophisticated laboratory equipment. In the earlier years of telomere research, a simple method named terminal restriction fragment analysis (TRF) emerged as one of the main and most accurate methods for telomere length analysis [12,13,14]. The method is based on genomic DNA digestion using specialized restriction enzymes that cut frequently throughout the genome, but not inside telomeric sequences. Digested DNA is subsequently separated by molecular weight using agarose gel electrophoresis, transferred to a nylon membrane, and visualized using radioactive or fluorescently labeled probes [15,16]. Some of the main advantages of the TRF method include the ability to measure the telomere length distribution in absolute values in kilobases (kb) and being reproducible and not prohibitively expensive. For many laboratories, telomere length measurement using the TRF protocol is still the gold standard method, and, over the years, various improvements have been made to enhance the detection and applicability [17,18].

**Figure 1:**
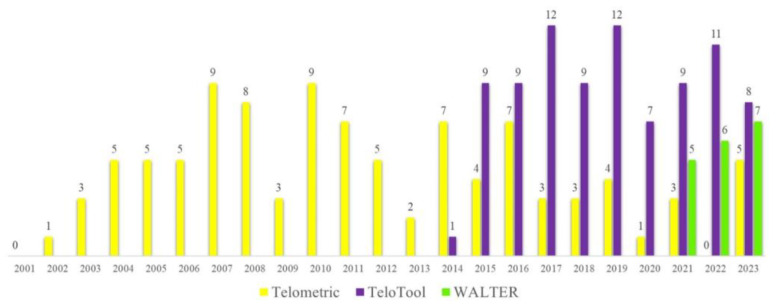
**Citations per year for the three telomere length measurement tools: Telometric, TeloTool, and WALTER.** The number of citations for the three original articles describing each method [19,20,23] was obtained from the Google Scholar database (accessed on 29 August 2023), and each of the papers was manually verified for the use of the corresponding method in calculating telomere length from TRF blots.

The classical TRF method usually produces a smear representing the area of the Southern blot where the specific oligonucleotide probe hybridizes to the telomeric DNA. This smear needs to be further quantified to obtain mean telomere length values for individual samples. Various software packages for the estimation of the telomere length from Southern blots have been developed to assist in the quantification of the telomere signals. One of the earliest specialized programs developed over 20 years ago to analyze TRF images was Telometric [19], which is still being extensively used in telomere research (Figure 1). However, Telometric calculates TRF values assuming a normally distributed intensity profile, often leading to substantially distorted median values. Furthermore, for very long telomeres over 12 kb, Telometric underestimates their lengths by up to 2 kb [20], reducing its feasibility for analysis in long-telomere species. In 2014, a new software named TeloTool (version 1.3.0.0) was developed, which fits the telomeric signal with a Gaussian function, making it particularly useful for samples with a unimodal distribution of the telomere-specific signal [20]. Over the years, TeloTool has been successfully used to examine the telomere length’s association with reproductive aging [21] and to evaluate telomere elongation processes specific to the alternative lengthening of telomeres (ALT) mechanism, which elongates telomeres in up to 15% of human cancers [22].

Finally, the most recently developed telomere length analysis tool, WALTER, is an online application that converts scanned TRF images into digital profiles consisting of telomere-specific signals and markers, allowing the analysis of signals with a non-unimodal distribution of the telomere intensity profiles [23]. WALTER appears to be particularly well suited for telomere dynamics studies, including the analysis of telomere length changes in human cell lines. Specifically, Mannherz and Agarwal (2023) [24] recently used WALTER to accurately measure the telomere length in 293T cells transduced with shRNA targeting the *SAMHD1* gene. Similarly, WALTER is also suitable for the analysis of developmental changes in telomere length, including age-related telomere attrition in a human brain structure called the putamen [25]. The main features of the TeloTool and WALTER telomere length analysis methods are summarized in Supplemental Table S1. 

We have previously measured the telomere length in 653 *A. thaliana* accessions and, through a genome-wide association study (GWAS), discovered several significant SNPs, including one inside the *TERT* gene encoding the catalytic subunit of telomerase [26]. The telomere length dataset for this study was generated using the TeloTool method [20]; however, a recent comparison of telomere length analysis tools indicated that the WALTER program may provide a better means to analyze data for *A. thaliana* samples, as TeloTool tends to overestimate the telomere length [23]. Since our laboratory is currently engaged in several large-scale quantitative telomere length screens, for which many samples were analyzed with TeloTool prior to the publication of the WALTER method, it was necessary to evaluate the applicability of both methods to large-scale genomic screens. Specifically, we wished to test whether the two analysis tools produce similar results that can be used in combination with each other when (1) analyzing DNA samples from hundreds of Arabidopsis genotypes for GWAS screens, and (2) evaluating the telomere length in individual T-DNA mutants of candidate genes as part of the follow-up molecular genetic tests.

Here, we show that TeloTool substantially overestimates the telomere length in comparison to WALTER, especially for values over 4500 bp. However, both programs produce comparable results for telomeres in the 2100–3500 bp range, indicating that the molecular analysis of most Arabidopsis T-DNA mutants of putative telomere biology genes can be performed equally well by both programs. Interestingly, the choice of telomere length analysis tool affects the outcomes of GWAS screens: the TeloTool dataset produces more significant SNPs than the WALTER dataset, although GWAS can identify the most significant hits using data from both datasets equally well. Collectively, these results indicate that data generated with different telomere length measurement tools can substantially influence downstream genomic and genetic screens.

## 2. Results

### 2.1. TeloTool—WALTER Comparison: General Differences and Similarities in Analyzing a Large-Scale Dataset

**Figure 2:**
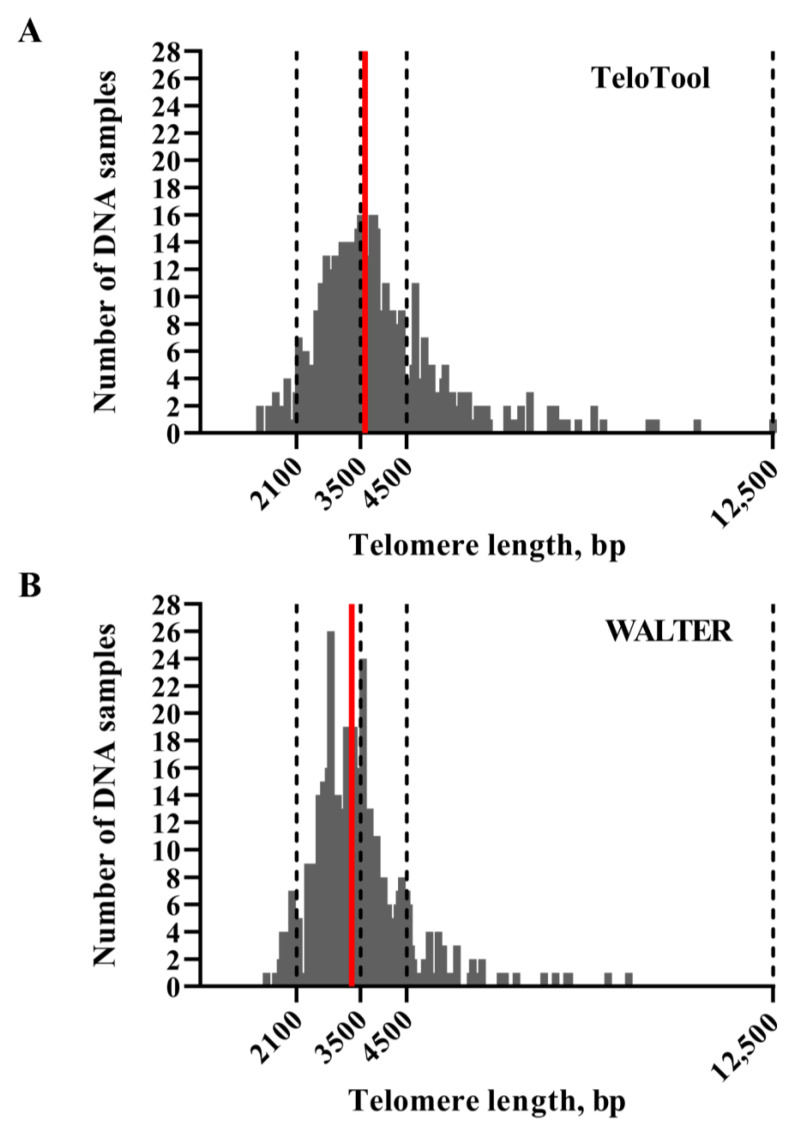
**Distribution of mean TRF values in Arabidopsis accessions used for GWAS analysis.** Telomere length values as calculated by TeloTool (**A**) and WALTER (**B**) programs are grouped into 50 base pair (bp) intervals, and the number of individual DNA samples falling into each interval are plotted against telomere length, sorted from shortest to longest. Red lines indicate median telomere length for each dataset. Dotted lines indicate limits of telomere length intervals selected for further analysis.

We first assessed how much the mean telomere length (mean TRF) data calculated by the TeloTool and WALTER programs differed from each other. We utilized telomere length data obtained through TRF blots for 537 Arabidopsis accessions (584 individual TRF measurements) (Supplemental Data S1). This dataset was smaller than the one used in our earlier study (653 accessions, [26]), and only included accessions for which TRF blots were performed in our laboratory. The distributions of the mean TRF values for this 537-accession dataset as measured by TeloTool and WALTER were plotted as bar graphs (Figure 2). The mean TRF values as calculated by TeloTool ranged from 1313 bp in the Ak-1 accession to 12,546 bp in the Mh-0 genotype, with a median length of 3592 bp (Figure 2A). For the WALTER analysis, mean TRF values ranged from 1451 bp in Hov1-10 to 9359 bp in the Wc-2 accession, with a median length of 3305 bp (Figure 2B). The overall distribution of the mean TRF values in the WALTER dataset was narrower than in the TeloTool dataset (Figure 2). Overall, TeloTool calculations on average provide higher values (longer telomere length) than those calculated with WALTER. We performed a paired Wilcoxon signed rank test with continuity correction to evaluate the difference between the estimates given by the two tools, which was statistically significant (*p* < 2.2 × 10^−16^).

**Figure 3:**
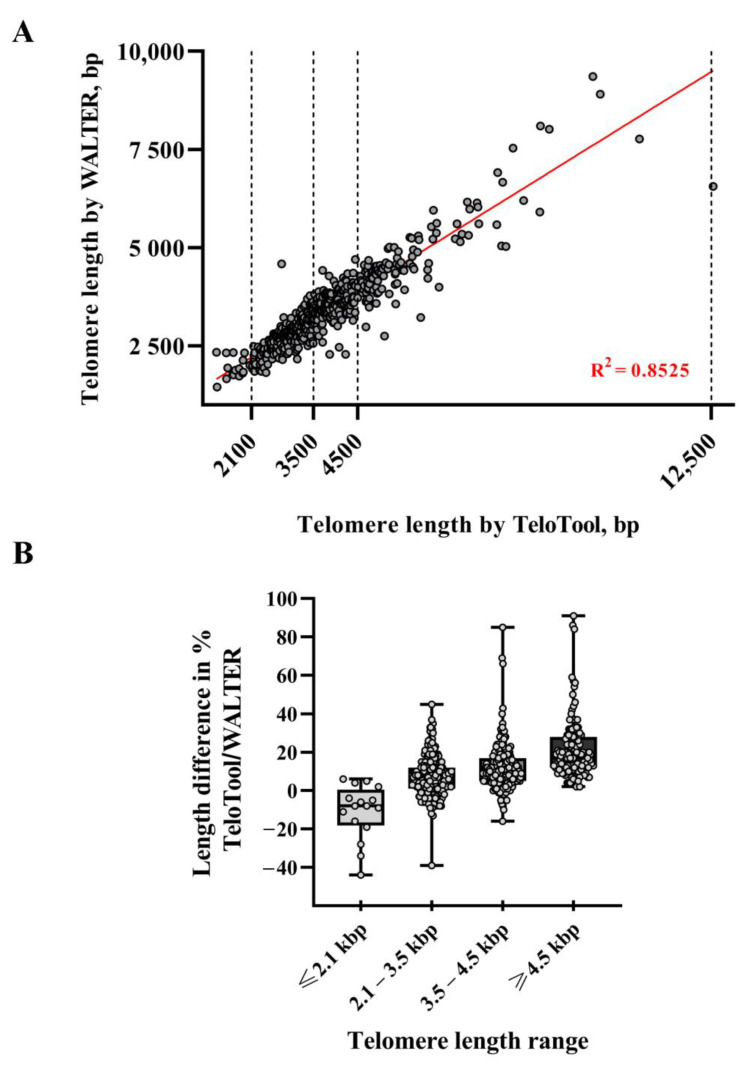
**Analysis of differences in telomere length measurements between TeloTool and WALTER programs**. (**A**) Length values for TeloTool and WALTER measurements are plotted for each DNA sample. Dotted lines indicate limits of selected telomere length intervals. The trend line is shown in red. (**B**) Box plots of the percent differences between TeloTool data and WALTER data for each telomere length range, determined as (TeloTool–WALTER)/WALTER × 100%. Whiskers indicate min to max range; points indicate the percent difference for each individual sample.

To further the TeloTool–WALTER comparison, we next calculated the overall correlations between the two datasets, which were relatively high: 0.9 (Spearman’s rho) and 0.92 (Pearson’s r) (Figure 3A). We then broke the data down into specific telomere length intervals, defined as short (≤2100 bp), medium (2101–3500 bp range), long (3501–4500 bp), and very long (≥4501 bp). The strongest correlations between the TeloTool and WALTER values were observed in the medium telomere length interval 2101–3500 bp (Spearman’s rho is 0.76 and Pearson’s r is 0.74) and in the very long telomere interval ≥4501 bp (Spearman’s rho is 0.77 and Pearson’s r is 0.84) (Supplemental Figure S1B,D). The correlation for the long telomere interval 3501–4500 bp was weaker (0.46 for Spearman and 0.41 for Pearson), while no statistically significant correlation between the TeloTool and WALTER values was observed for the ≤ 2100 bp interval (0.07 for Spearman and 0.08 for Pearson) (Supplemental Figure S1A,C).

We also compared the absolute differences between the TeloTool and WALTER values. In support of the notion that TeloTool tends to overestimate particularly long telomeres (in comparison to WALTER), for all analyzed telomeres above 4.5 kb (123 individual DNA samples for 111 accessions), the TeloTool values were higher than the WALTER values, with several TeloTool measurements being up to 91% higher than the WALTER values (Figure 3B, Table 1). For the second group of DNA samples with a telomere length in the long range (3501–4500 bp), the overestimation of the telomere length by TeloTool was also observed, with the mean difference between values in this telomere length interval being at 12.01% (Table 1). For the telomere length data in the medium range (2101–3500 bp), we observed the greatest correlation between the TeloTool- and WALTER-calculated values, with the mean difference between the two datasets being only 7.40% (Figure 3B, Table 1). Finally, for the short (≤2100 bp) telomere length range, the WALTER values were on average slightly longer than the TeloTool values (mean difference is −10.94%) (Table 1), although this effect was largely driven by 3 out of 16 DNA samples in this range (Figure 3B), and their removal from the analysis decreased the mean difference in this size range to −5.30%. Taken together, our data indicate that TeloTool- and WALTER-generated data overall correlate well but display the most divergence in the shortest telomere length range ≤ 2100 bp, while showing the greatest correlation in the 2101–3500 bp range.

### 2.2. Telomere Length Estimates by TeloTool or WALTER Methods Can Affect GWAS Outcomes

To evaluate how the telomere length values extracted from TRF blots by different programs can influence the results of large-scale genomic assays, we performed separate GWAS analyses using data generated by TeloTool and WALTER. GWAS was performed using the GWA-Portal (https://gwas.gmi.oeaw.ac.at, accessed on 3 September 2023), a user-friendly and interactive web application for running Arabidopsis GWAS studies [27]. Data were analyzed through the standard pipeline using the following parameters: Imputed Fullsequence Dataset for Arabidopsis genotypes (TAIR 9) and LOG transformation for telomere length data.

**Figure 4:**
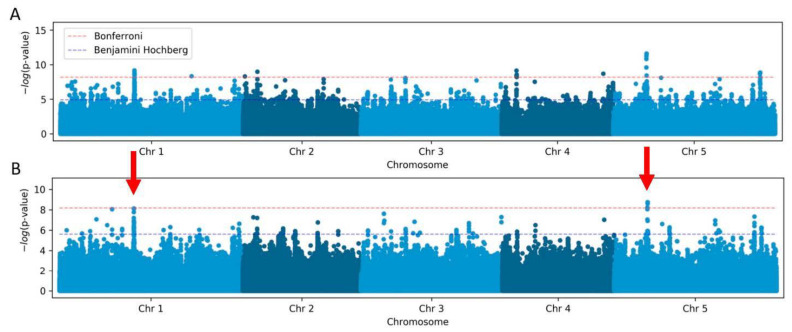
**GWAS of *A. thaliana* telomere length variation using the linear regression (LM) method.** Manhattan plot of the genome-wide *p*-values indicating the strongest associations between the five Arabidopsis chromosomes and telomere length data obtained from TeloTool (**A**) and WALTER (**B**). Red dotted lines indicate the Bonferroni-corrected significance threshold (α = 0.05). The GWAS-significant regions discovered with both the TeloTool and WALTER datasets are indicated by red arrows.

To initiate our analysis, we first performed GWAS with TeloTool-generated data using the simple linear regression (LM) method, which revealed ten genomic regions with single-nucleotide polymorphisms (SNPs) significantly associated (after Bonferroni correction) with the telomere length (Figure 4A). In contrast, the analysis of the WALTER dataset using the same method revealed only three genomic regions significantly associated with the telomere length (Figure 4B). Importantly, two of the most significant SNPs with the highest *p*-values were detected in both analyses. One of these significant SNPs, on the chromosome 5 position 5,538,242, is located inside the *TERT* gene At5g16850 (Table 2) and represents the same SNP that was identified in our earlier study [26]. The identification of the *TERT* gene polymorphism in this and the previous studies, as well as when using both the TeloTool and WALTER datasets, implies that both telomere length analysis tools are suitable for the identification of the most significant hits in large-scale genomic studies.

The second significant SNP detected with both the TeloTool and WALTER datasets is located on the chromosome 1 position 12,379,845 in the intergenic region near the promoter of the gene At1g34042 (Table 2), which appears to be highly expressed in flowers, roots, and seeds and encodes a small hypothetical protein. Other genes located in the vicinity of this SNP include *Tryptophan Aminotransferase Related 3* (*TAR3*), *Ribosomal Protein S13*, and *UDP-RHA/UDP-GAL Transporter 6* (*URGT6*). The presence of the ribosomal protein S13 is particularly intriguing, since previous genome-wide assays have implicated ribosome biogenesis factors in telomere length control [28].

Eight other significant SNPs identified in the TeloTool dataset have lower *p*-values and are distributed across all five Arabidopsis chromosomes, mostly in intergenic regions or inside protein-coding genes (Table 2). The three notable examples are SNPs located on chromosome 4, position 2,463,357 (intron of At4g04870 gene, which encodes Cardiolipin synthase) and position 16,935,117 (missense nucleotide change aTg/aAg in At4g35733 gene, leading to M231K substitution in F-box SKIP23-like protein), and on chromosome 3, position 7,407,370 (missense nucleotide change Gga/Aga in At3g21120 gene, leading to G349R substitution in a little-characterized F-box protein).

One additional significant SNP identified using the WALTER dataset is located on chromosome 1, position 8,743,486 (intron of At1g24706 gene, which encodes THO2, a component of the putative Arabidopsis THO/TREX complex). Interestingly, with the exception of the polymorphism located inside the *TERT* gene, none of the other 10 significant SNPs from both GWAS analyses correspond to SNPs identified in our previous study using a larger dataset of 653 accessions [26]. However, two significant SNPs (chromosome 2, positions 2,422,473 and 356,311, from the TeloTool dataset) are located in a quantitative trait loci (QTL) interval on chromosome 2 that was previously identified in a telomere length mapping study using a Pro-0/Col-0 recombinant inbred line population [29].

We next repeated our GWAS analyses with the accelerated linear mixed model (AMM), which is the only method available in the GWA-Portal that accounts for the population structure and thus should work better in identifying the loci of complex traits for species confounded by the population structure, like Arabidopsis [30]. With the AMM method, the analysis of both the TeloTool and WALTER datasets identified the same significant SNP on chromosome 5, position 5,538,242, that is located inside the *TERT* gene (Supplemental Figure S2). Additionally, GWAS analysis using the TeloTool dataset identified two more significant SNPs (Table 2). One is located very close to the telomere on chromosome 3, position 23,295,214 (promoter of At3g63030 gene, which encodes METHYL-CPG-BINDING DOMAIN 4 protein), and one is located in the intergenic region on chromosome 1, position 21,870,431, between genes At1g59530 (encoding BASIC LEUCINE-ZIPPER 4 protein) and At1g59540 (encoding a kinesin-like protein). No other significant SNPs were discovered with the AMM method for either the TeloTool or WALTER dataset.

Overall, the results of our GWAS analyses suggest that data obtained using the TeloTool or WALTER programs will provide partially overlapping but not identical results. Specifically, GWAS analyses with both datasets identified the same one (AMM) or two (LM) most significant SNPs with the highest *p*-values, but all additional SNPs with lower *p*-values differed between the two analyses. Furthermore, GWAS analysis performed with the TeloTool dataset revealed more significant SNPs than was the case for the WALTER dataset, regardless of the GWAS algorithm used. We conclude that both telomere length analysis tools can be used to identify the most significant hits, but less significant candidates will likely differ between the two datasets. We further suggest that the higher number of significant SNPs identified with the TeloTool dataset can likely be attributed to the overestimation of the telomere length by this program, in comparison to WALTER.

### 2.3. Comparison of TeloTool and WALTER Datasets Generated for the Arabidopsis T-DNA Mutant Screen

**Figure 5:**
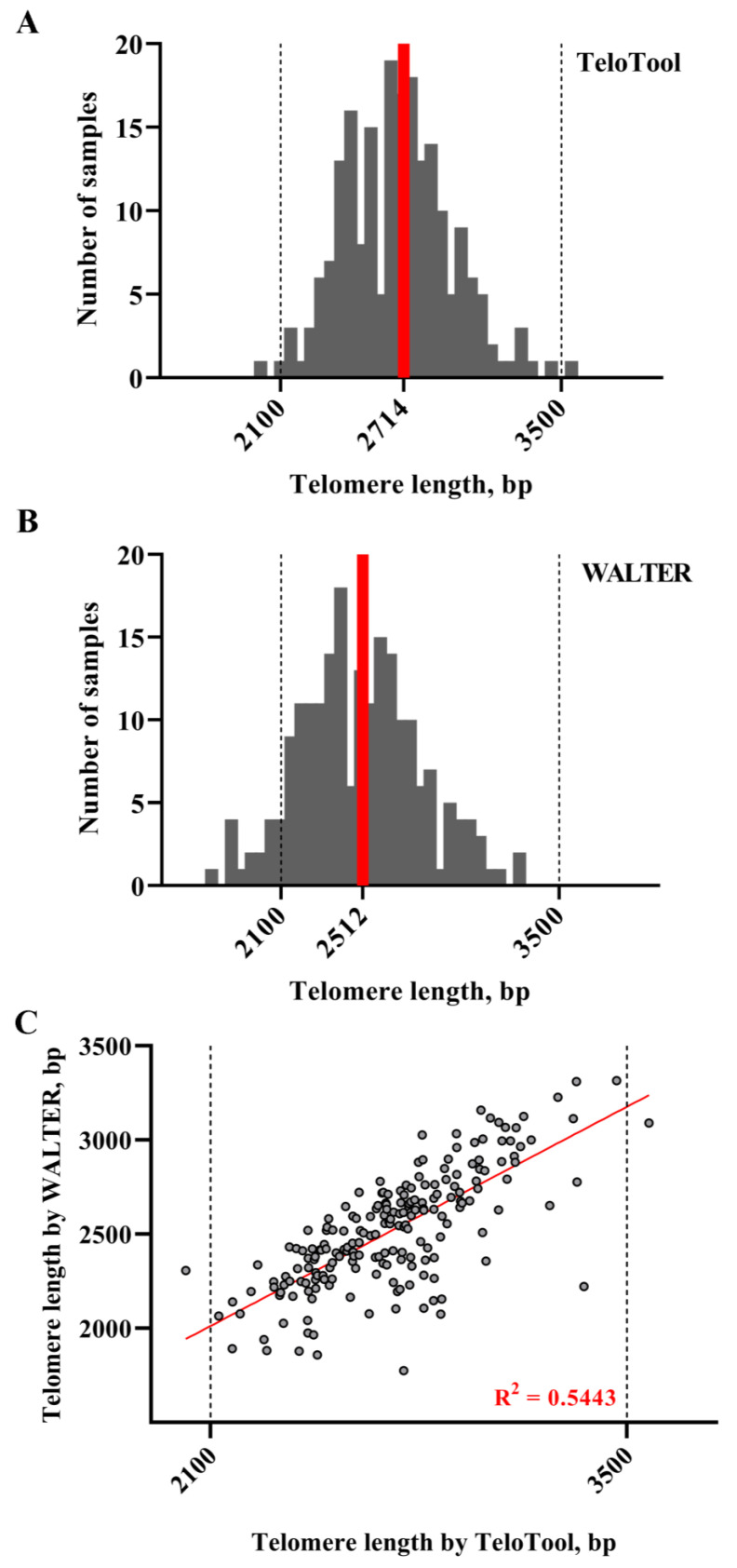
**Distribution of mean TRF values in Arabidopsis T-DNA mutant plants.** Telomere length values as calculated by TeloTool (**A**) and WALTER (**B**) programs are grouped into 50 bp intervals, and the number of individual DNA samples falling into each interval is plotted against telomere length, sorted from shortest to longest. Red lines indicate median telomere length for each dataset. Dotted lines indicate the medium telomere length range (2100–3500 bp) characteristic of most Arabidopsis T-DNA mutants. (**C**) Length values for TeloTool and WALTER measurements are plotted for each DNA sample. The trend line is shown in red.

We next examined the utility of the WALTER and TeloTool telomere length measurement methods for the screening of a large collection of *A. thaliana* T-DNA mutants generated in the reference Columbia genotype [31]. In total, TRF data for 205 individual DNA samples were analyzed, which included 22 replicates of the reference Col-0 accession, 51 individual T-DNA mutant lines with two or more biological replicates for each, and 14 T-DNA lines with only a single biological replicate (Supplemental Data S2). As was expected for T-DNA mutants generated in the same genetic background, the overall distribution of mean TRF values calculated using TeloTool was much narrower (Figure 5A) than that observed for natural Arabidopsis accessions and ranged from 2017 to 3574 bp, with the median length of 2714 bp. This telomere length range effectively corresponds to the “medium” group of telomere lengths described for the GWAS samples. An analysis of the same TRF blots with WALTER produced a similar profile (Figure 5B), although the overall distribution of TRF values was shifted to the left in comparison to the TeloTool data and ranged from 1776 to 3315 bp, with the median length of 2520 bp. The correlation coefficients between the TeloTool and WALTER values for the telomere length of T-DNA mutant lines were also relatively high, 0.73 for Spearman and 0.74 for Pearson (Figure 5C). Overall, similar to the situation with different Arabidopsis accessions, we observed a general trend for TeloTool to overestimate the telomere length compared to WALTER. However, we infer from this analysis that when proper wild-type controls are included in the study, either program can be used equally well to analyze the telomere length phenotypes of mutants of Arabidopsis gene candidates discovered through GWAS or other large-scale genomic assays.

## 3. Discussion

Terminal restriction fragment analysis is a powerful and efficient way to measure telomere length in a number of species and populations. While different methods have been extensively used in the past to extract telomere length information from TRF gels, such as the GelQuant (biochemlabsolutions.com, V 1.7.8) [32] or Multi Gauge V3.0 package [33], the development of several specialized tools dedicated to telomere length quantification [19,20,23] has marked a major step forward towards normalizing and comparing the results obtained from different gels and even from different research laboratories.

TeloTool and WALTER utilize contrasting approaches to calculate the telomere length, and each method has its own advantages and drawbacks. Specifically, previous observations indicated that TeloTool can overestimate telomeres, while WALTER can potentially allow for a more subjective calculation [23]. Using hundreds of individually analyzed DNA samples, we have compared the applicability of both methods to downstream large-scale genomic assays. Our findings using data for 537 natural Arabidopsis accessions confirmed previous observations [23] that the TeloTool software can indeed overestimate the telomere length, with the important additional clarification that this bias especially affects very long telomeres above 4500 bp. For the shortest telomere range below 2100 bp, we also observed substantial variation between TeloTool- and WALTER-generated data, but it should be noted that due to the peculiarity of the hybridization kinetics, short telomeres (2 kb or less) are generally difficult to measure with any quantification method, and especially with TRF assays [11]. Additionally, we noticed that the samples in our analysis that showed the greatest difference between TeloTool and WALTER values often came from TRF gels that were characterized by reduced quality of the telomeric DNA signal (minor signs of degradation, weak signal, stains, bubbles). While some of these technical challenges are unavoidable when conducting the large-scale screening of hundreds of natural accessions or T-DNA mutants, general improvements in the TRF technique will, to some extent, minimize the differences in the values calculated by the two programs.

Our findings also indicate that data generated with both TeloTool and WALTER can be used to detect the most significant SNPs in GWAS screens. The identification of the previously described (through a larger 653-accession study by [26] significant SNP inside the *TERT* gene can be viewed as an internal positive control highlighting the general applicability of both methods to GWAS assays. However, the use of telomere length data generated by the TeloTool program resulted in the detection of many more statistically significant SNPs than was obtained in the case of the WALTER dataset, regardless of the GWAS method used (AMM or LM). Only two overlapping significant SNP hits were identified using data from both TeloTool and WALTER, suggesting that WALTER provides a more conservative approach to GWAS mapping. We speculate that this substantial variability in GWAS results can be largely explained by differences in measuring the most extreme telomere length phenotypes (longer than 4.5 kb and below 2.1 kb).

For the T-DNA mutant screening experiment, our analysis indicated that the TeloTool and WALTER values in the size range of 2100–3500 bp were relatively similar to each other, suggesting that both programs can be used interchangeably for telomere length analysis, if all proper wild-type controls are included. Since we also detected some degree of variation even between Col-0 wild-type plants, we recommend that, whenever possible, the TRF analysis of homozygous T-DNA mutant plants should be performed in comparison with their corresponding wild-type siblings, and not with unrelated wild-type plants. This is also important in the context of comparing data with studies from other laboratories, especially when telomere length calculation is carried out with a different program.

## 4. Materials and Methods

### 4.1. Plant Materials and Growth Conditions

For the GWAS experiment, seeds for the set of *A. thaliana* genotypes from the 1001 Genome Project were purchased from the Arabidopsis Biological Resource Center (ABRC CS78942). A total of 584 DNA samples were run on TRF gels for telomere length analysis, representing 537 accessions from the CS78942 set, including 43 accessions with more than one biological replicate (individual plants). The 537-accession subset included previously published telomere length data for 424 *A. thaliana* accessions [26], as well as new data for 113 additional accessions included in this study (Supplemental Data S1).

For the T-DNA mutant screen, seeds for the wild-type accession Col-0 (CS6673) and individual T-DNA mutants (Supplemental Data S2) were obtained from ABRC. A total of 205 DNA samples were run on TRF gels for telomere length analysis, representing one or more biological replicates of 65 individual T-DNA lines and the wild-type control Col-0. Seeds were sown into a mixture of three parts Promix BX mycorrhizae soil, one part Profile Field and Fairway calcined clay, and one part Turface medium stabilizer, and plants were grown as described earlier [26]. Plant tissue for TRF analysis was collected at the 5-week stage.

### 4.2. Telomere Length Measurement

Genomic DNA was extracted from individual whole plants and digested with the restriction enzyme Tru1I (Fermentas, Hanover, MD, USA) as previously described [34]. The [32P] 5′-end labeled or 5′-DIG-(T_3_AG_3_)_4_ oligonucleotides were used as probes [18,28]. Radioactive signals were scanned with a Pharos FX Plus Molecular Imager (Bio-Rad, Hercules, CA, USA), and nonradioactive signals were scanned with a GBox-F3 Imager (Syngene, Frederick, MD, USA). Images were visualized with the Quantity One v.4.6.5 software (Bio-Rad), and mean telomere length values (mean TRF) were calculated using the TeloTool program (v 1.3.0.0) [20] or the WALTER program (v 2.0) [23]. TRF gels were run by different researchers, but all calculations with both TeloTool and WALTER were performed by the same person.

### 4.3. Genome-Wide Association Study

The GWA-Portal (https://gwas.gmi.oeaw.ac.at, accessed on 3 September 2023) web application was utilized to perform the Arabidopsis GWAS studies [27]. Telomere length phenotypes (Supplemental Data S1 and S2) were uploaded as a comma-separated values (csv) file. Analysis was conducted following the general guidelines [27] with the “Imputed Fullsequence” genotype dataset (2029 genotypes with ~10 million SNPs), which represents a combined dataset of the 250K SNP dataset and the 1001 genomes dataset using imputation. Upon performing the LOG transformation of the telomere length for both the TeloTool and WALTER datasets, GWAS was conducted with the linear regression (LM) method and with the accelerated linear mixed model (AMM).

### 4.4. Statistical Analysis

Statistical analysis was carried out with the GraphPad Prism 8 software (version 8.0.1) (San Diego, CA, USA). Trend line analysis was performed using linear regression parameters. The number of DNA samples, minimum and maximum differences, and median and mean values of differences were calculated using the descriptive statistics parameters. Correlation coefficients for the Pearson and Spearman methods along with their statistical significance were calculated using the R software (version 4.3.2, cor.test() function).

## 5. Conclusions

In conclusion, our study provides several important recommendations for the evaluation of large-scale telomere length datasets for genomic studies and for the analysis of individual mutants with deregulated telomere length homeostasis. First, when analyzing DNA samples from hundreds of Arabidopsis accessions, TeloTool and WALTER should not be used in combination with each other for telomere length calculations, as each program calculates the telomere length differently, especially in the very long range of over 4500 bp. If a more conservative approach to GWAS is desired, one should choose WALTER, but if a more extended candidate list is expected, TeloTool may be the more appropriate program to choose. However, when evaluating the telomere length in individual T-DNA mutants of candidate genes following extensive genomic screens, either program will be efficient in analyzing the telomere length. Although both the TeloTool and WALTER programs were developed by research groups primarily working with the model plant *Arabidopsis thaliana* [20,23], these programs have quickly gained popularity for telomere length analysis in many other classical and emerging plant models [35], as well as for the analysis of non-plant telomeres, including those in human cells, cancer cell lines, green algae, and Trypanosomes [17,24,25,36,37,38]. Thus, our results may be relevant for telomere biology studies and the functional analysis of candidate genes in other systems, including large-scale genomic screens in other models and in humans.

## Figures and Tables

**Table 1:** Statistical differences between TeloTool and WALTER datasets for Arabidopsis accessions.

Telomere Length Range *	≤2100 bp	2101–3500 bp	3501–4500 bp	≥4501 bp
Number of DNA samples	16	250	195	123
Number of accessions	15	232	179	111
Minimum difference, %	−44.00	−39.00	−16.00	2.00
25% Percentile	−18.25	1.000	5.000	12.00
Median difference, %	−8.00	7.000	10.00	17.00
75% Percentile	0.50	12.00	17.00	28.00
Maximum difference, %	6.00	45.00	85.00	91.00
Mean difference, %	−10.94	7.40	12.01	21.68

* Telomere length ranges are defined based on the TeloTool dataset. Percent differences between TeloTool and WALTER values calculated as (TeloTool-WALTER)/WALTER × 100%.

**Table 2:** GWAS-significant SNPs for telomere length in the TeloTool and WALTER datasets.

SNP	Chromosome	Position	*p*-Value	Major Allele	Minor Allele	maf	Effect
**TeloTool (LM)**							
5:5538242 *	5	5538242	11.61	T	C	0.19	3’UTR_3 At5g16850
1:12379845 *	1	12379845	10.46	G	A	0.028	Intergenic
4:2463357	4	2463357	9.13	C	T	0.046	INTRON At4g04870
2:2422473	2	2422473	8.98	G	A	0.059	Intergenic
5:24491588	5	24491588	8.86	G	A	0.055	Intergenic
4:18303636	4	18303636	8.78	C	T	0.028	Intergenic
4:16935117	4	16935117	8.7	T	A	0.04	MISSENSE aTg/aAg M231K At4g35733
1:21870431	1	21870431	8.32	T	G	0.038	Intergenic
2:356311	2	356311	8.30	T	A	0.036	Intergenic
3:7407370	3	7407370	8.06	G	A	0.104	MISSENSE Gga/Aga, G349R At3g21120
**WALTER (LM)**							
5:5538242 *	5	5538242	8.73	T	C	0.19	3’UTR_3 At5g16850
1:12379845 *	1	12379845	10.01	G	A	0.028	Intergenic
1:8743486	1	8743486	8.06	A	G	0.04	INTRON At1g24706
**TeloTool (AMM)**							
5:5538242 *	5	5538242	11.61	T	C	0.19	3’UTR_3 At5g16850
3:23295214	3	23295214	8.66	T	G	0.03	Promoter of At3g63030
1:21870431	1	21870431	8.08	T	G	0.038	Intergenic
**WALTER (AMM)**							
5:5538242 *	5	5538242	9.0	T	C	0.19	3’UTR_3 At5g16850

* Asterisks indicate significant SNPs identified with both datasets.

## Data Availability

The data that support the findings of this study are available in the Supplementary Materials of this article.

## Supplementary Materials

The supporting information can be downloaded at: https://www.mdpi.com/article/10.3390/ijms242417194/s1.
