# Supplementary material for: Comparative Application of Terminal Restriction Fragment Analysis Tools to Large-Scale Genomic Assays
Source: Int J Mol Sci. 2023 Dec 6;24(24):17194. doi: 10.3390/ijms242417194 (PMC10742804; doi:10.3390/ijms242417194)
Supplement: Supplementary file 1 [file ijms-24-17194-s001.zip › Supplemental Material.pdf]

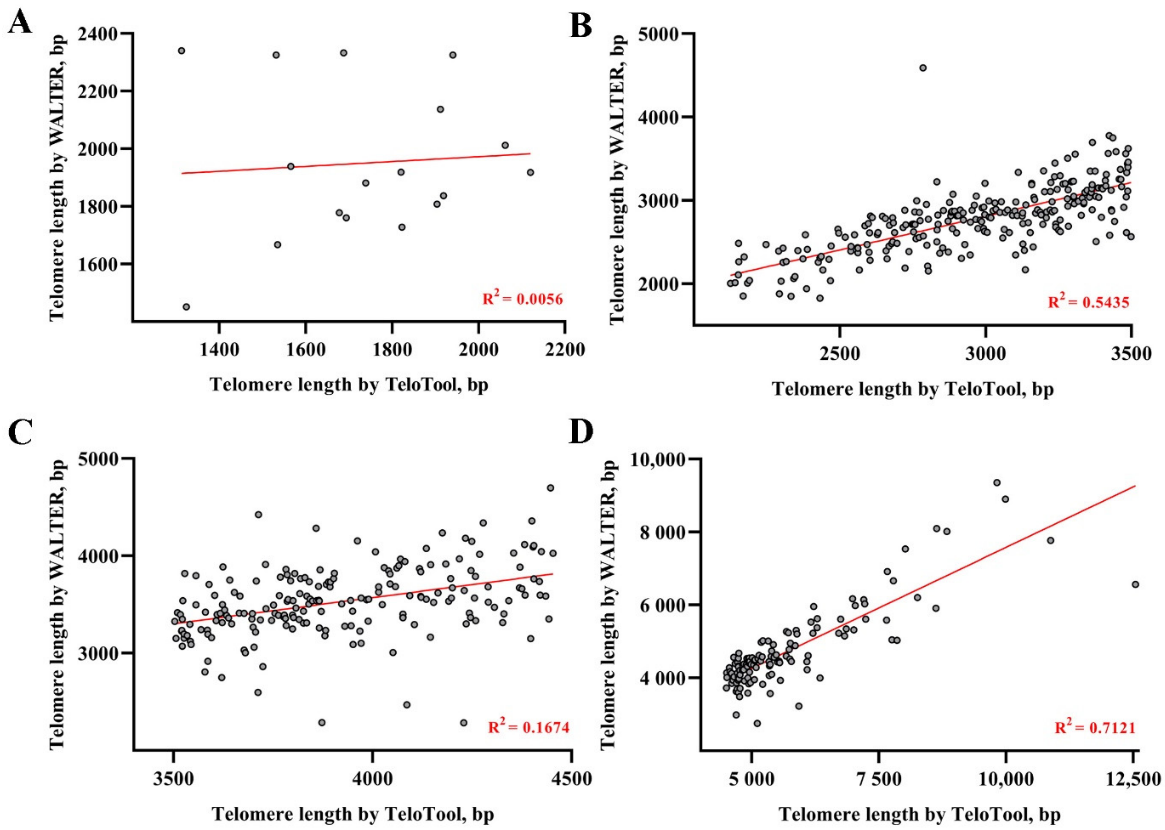

Supplementary Figure S1. Analysis of differences in telomere length measurements between TeloTool and WALTER programs for selected length intervals. Telomere length values for (**A**) short (≤ 2,100 bp), (**B**) medium (2,101-3,500 bp), (**C**) long (3,501-4,500 bp), and (**D**) very long (≥4,501 bp) intervals are plotted. Trend lines are shown in red.

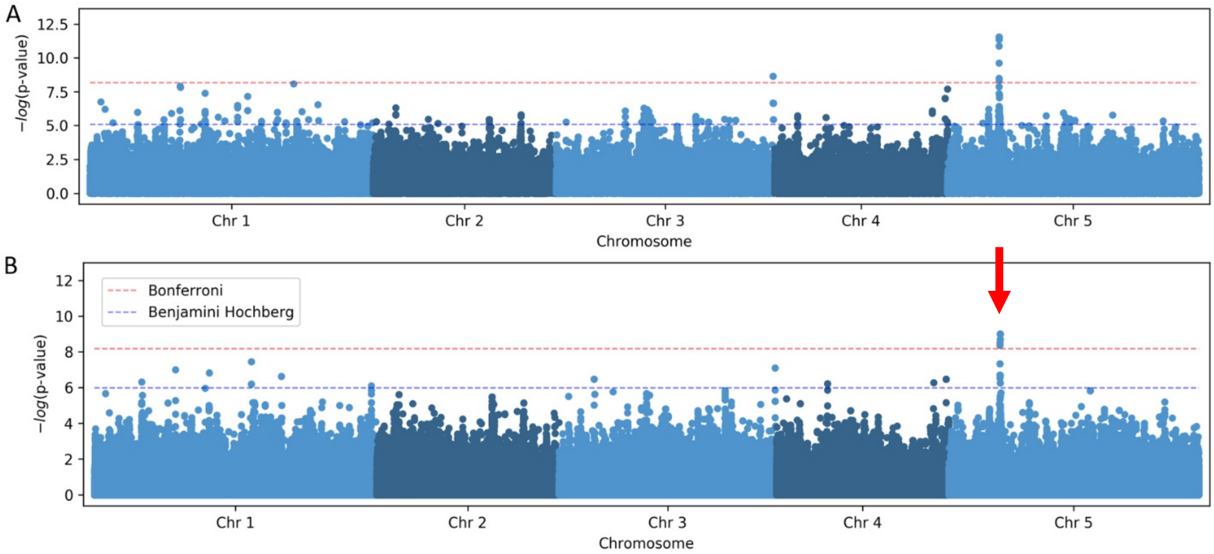

Supplementary Figure S2. GWAS of *A. thaliana* telomere length variation using the accelerated linear mixed model (AMM) method. Manhattan plot of the genome-wide *P*-values indicating the strongest associations between the five Arabidopsis chromosomes and telomere length data obtained from TeloTool (**A**) and WALTER (**B**). Red dotted lines indicate the Bonferroni-corrected significance threshold (α = 0.05). The GWAS-significant region inside the *TERT* gene discovered with both the TeloTool and WALTER datasets is indicated by a red arrow.

Supplemental Table S1. Notable features of TeloTool and WALTER programs.

**Program name**	**TeloTool**	**WALTER**
Reference	Gohring et al., 2014, doi:10.1093/nar/gkt1315	Lyčka et al., 2021, doi:10.1186/s12859-021-04064-0
Download requirements	Both TeloTool and MATLAB are required.	Works either over internet (requires uploading two files in xls and html format) or offline (requires downloading WALTER toolset v2.0 Portable)
Track recognition method	Automatic by the Otsu method	Manual (user marks a track)
Method for recognizing track width	Automatically recognized (can be adjusted using the 'Filter for Lane Width' button)	Manual (user marks the track width)
DNA size marker specification and fitting	$R^2_{adj} = 1 - \left(\frac{SS_{res}}{SS_{tot}}\right) * \left(\frac{N-1}{N-d-1}\right)$ R^2_{adj}- is adjusted coefficient of determination; d- is the degrees of freedom of the polinominal; SS_{res}-residual sum of squares; SS_{tot}- total sum of squares.	ANOVA hypothesis
Data analysis method	Data described by a Gaussian curve $f(x) = \frac{1}{\sigma\sqrt{2\pi}} * e^{-\frac{(x-\mu)^2}{2\sigma^2}}$ μ is the mean deviation of the Distribution; σ is the standard deviation of the distribution	$OD_{lt} = OD_{lb} + W(OD_{lmax} - OD_{lb})$ $OD_{rt} = OD_{rb} + W(OD_{rmax} - OD_{rb})$ OD_{lt}, intensity values for the left; OD_{rt}, intensity values for the right; OD_{lmax}, the maximum signal intensity within the sequence; OD_{lb}, the lowest intensity value within the sequence; OD_{rb}, the lowest intensity value within the sequence OD_{rmax}; W, a constant describing the width of the chosen area with its value set by

		default to 0.28 based on empirical testing.
Background correction	Three options are available:<br>1) The software 'trendline' function identifies the lowest values among the initial and final 10 pixels of wach lane. It then calculates a line connecting these points along the lane before deducing the trendline from the raw data;<br>2) the 'baseline' option calculates the mean between the two values in 1) and subtracts the mean from the original data;<br>3) The background of the gel can be defined manually by the user. The mean value of this area can then be subtracted from the raw data.	Background correction is based on the a linear model constructed from two points located to the left and right of the telomere-specific signal (similar to TeloTool, but area is much larger).
Image upload format	tiff	jpeg
Editing the image	Directly in the app	With ScanToIntensity
Area of signal intensity	Determined automatically	Determined automatically, but can be adjusted manually
Report file upload	In xls format	In HTML format
